# A Fading Suppression Method for Φ-OTDR Systems Based on Multi-Domain Multiplexing

**DOI:** 10.3390/s25082629

**Published:** 2025-04-21

**Authors:** Shuai Tong, Shaoxiong Tang, Yifan Lu, Nuo Yuan, Chi Zhang, Huanhuan Liu, Dao Zhang, Ningmu Zou, Xuping Zhang, Yixin Zhang

**Affiliations:** 1 College of Engineering and Applied Sciences, Nanjing University, Nanjing 210023, China; tongshuai@smail.nju.edu.cn (S.T.); 502022340011@smail.nju.edu.cn (S.T.); 522023340009@smail.nju.edu.cn (Y.L.); 522024340027@smail.nju.edu.cn (N.Y.); dg21340017@smail.nju.edu.cn (C.Z.); nzou@nju.edu.cn (N.Z.); 2 Key Laboratory of Intelligent Optical Sensing and Manipulation, Ministry of Education, Nanjing University, Nanjing 210093, China; lhh_ly@163.com; 3 Shenzhen Institute of Advanced Technology, Chinese Academy of Sciences, Shenzhen 518055, China; 4 Nanjing Fiber Technology Co., Ltd., Nanjing 211135, China; zdaonj@gmail.com; 5 School of Integrated Circuits, Nanjing University, Suzhou 215163, China; 6 Shenzhen Research Institute, Nanjing University, Shenzhen 518000, China

**Keywords:** phase-sensitive optical time domain reflectometry (Φ-OTDR), fading noise, spatial-domain multiplexing (SDM), polarization-domain multiplexing (PDM), frequency-domain multiplexing (FDM), spatial weighting, multi-domain multiplexing (MDM)

## Abstract

The phase-sensitive optical time domain reflectometry (Φ-OTDR) has been widely applied in various fields. However, due to fading noise, false alarms often occur; this limits its engineering applications. In this paper, a fading suppression method for Φ-OTDR systems based on multi-domain multiplexing (MDM) is proposed. The principles and limitations of existing suppression methods such as spatial-domain multiplexing (SDM), polarization-domain multiplexing (PDM), and frequency-domain multiplexing (FDM) are analyzed. The principle of MDM is explained in detail, and an experimental system is established to test the fading noise suppression capabilities of different parameter combinations of the PDM, FDM, and SDM methods. Experimental results show that it is difficult to comprehensively suppress fading noise with single-domain multiplexing. Through optimizations of different parameter combinations, MDM can comprehensively suppress fading noise by appropriately selecting the number of FDM frequencies, the spatial weighting intervals, and using PDM, thus obtaining the optimal anti-fading solution between performance and hardware complexity. Through MDM, the fade-free measurement is achieved, providing a promising technical solution for the practical application of the Φ-OTDR technology.

## 1. Introduction

Distributed fiber-optic sensing technology possesses both signal transmission and sensing capabilities, enabling the acquisition of tens of thousands of sensing points along a single fiber. It also features advantages such as electromagnetic interference immunity, high sensitivity, and wide detection range [1], making it a research hotspot. Phase-sensitive optical time domain reflectometry (Φ-OTDR) has become an important branch of it, standing out for its high sensitivity and fast response speed [2] for monitoring various physical parameters. Φ-OTDR is based on the Rayleigh backscattered signal (RBS), employing a highly coherent laser as the light source, which has been widely applied in various fields, including perimeter security monitoring [3,4], marine monitoring [5,6], and earthquake monitoring [7,8].

However, Φ-OTDR systems are often affected by fading noise, which has limited their application [9,10,11,12]. Typically, fading noise includes interference fading noise and polarization fading noise. Interference fading noise is caused by the inhomogeneity of the refractive index of the fiber core and the use of a narrow linewidth laser as the light source in Φ-OTDR systems. The presence of interference fading leads to random fluctuations in the amplitude of the RBS [9]. Polarization fading refers to the mismatch of polarization states between the local light and the RBS, which decreases the visibility of the intermediate frequency (IF) signal [10]. This fading may cause the amplitude of the IF signal to drop into a lower range, which leads to waveform distortion in the demodulated time-domain signal, a decrease in the signal-to-noise ratio (SNR), and, consequently, a reduction in the accuracy of monitoring.

The fading mentioned above always leads to false alarms, which cause many inconveniences to the application of Φ-OTDR systems in engineering. To suppress fading noise, researchers have explored many methods, such as spatial-domain multiplexing (SDM) [11,12,13,14], polarization-domain multiplexing (PDM) [10,15,16], and frequency-domain multiplexing (FDM) [17,18,19]. All these methods achieve fading noise suppression by multiplexing independent and uncorrelated channels.

SDM suppresses fading through the spatially independent distribution of the RBS. Three of the seven optical fiber cores were connected start-to-end in an S shape [11], and a strategy based on spectral similarity was used to process multiple independent signals. The distortion rate could be reduced from an average of 9.34% to less than 2% under 270 s of continuous operation. However, due to the series connection of multiple fiber cores, the frequency response and the dynamic range of the system were limited. Another way to implement SDM is to use a spatial weighting algorithm [12,13,14]. This is initially achieved through maximum amplitude selection [12] and direct averaging [13]. However, the phase consistency and the spatial resolution consistency of adjacent spatial regions are destroyed. Then, a spatial weighting algorithm based on the carrier-to-noise ratio (CNR) was proposed [14], reducing the probability of fading noise from 6.23% to 0.19%. However, when the weighting intervals increase, the spatial resolution inevitably decreases. Moreover, none of these methods can suppress the polarization fading noise.

PDM suppresses fading through the polarization state-independent distribution of the IF signal. By optimizing complementary polarization state measurements, the fading was suppressed [10]. The statistical characteristics of location-related intensity distribution in Φ-OTDR were studied [15], and the probability density functions in the cases of polarization mismatch and polarization match were given. When simultaneously detecting vibration signals of 500 Hz and 800 Hz, compared with the traditional coherent detection Φ-OTDR, the SNR of the polarization diversity detection was increased by 10.9 dB and 8.65 dB, respectively. The orthogonal SOP pulse pair (OSPP) method was used to generate orthogonal polarization state pulses [16]. By summing the IF signals of the orthogonal polarization states, the polarization fading noise was effectively suppressed, enabling the disturbance events to be clearly resolved. However, the summing operation reduces the effective pulse repetition frequency by half, thus causing the frequency response of the system to be halved as well. However, these methods cannot suppress the interference fading noise. Therefore, fading suppression remains incomplete.

FDM suppresses fading through the frequency-independent distribution of the RBS. When using probe lights with different frequencies, it is possible to avoid the fading phenomenon because the locations of the fading areas vary for different independent probe frequencies. Based on the differences in the frequency distribution of the RBS, the fading noise can be suppressed by multiplexing the different probe frequencies. Three different probe frequencies were used for an Φ-OTDR system to select the optimal probe signal at any time [17]. It predicted the occurrence of fading before it actually happened. The fading probability was reduced to 1.15%. In this case, polarization fading was not taken into account. A method based on FDM and optimal tracking was proposed to predict the optimal phase signal and achieved continuous suppression of fading [18]. The distortion caused by fading was suppressed to 1.26%. The enhancement of the SNR in a multifrequency Φ-OTDR system had an analytical relationship of $\sqrt{n}$ between the enhancement of the SNR and the number of probe frequencies used [19]. The higher the suppression of fading noise, the greater the number of frequencies that need to be multiplexed. As the number of probe frequencies increases, more frequency signals need to be transmitted within a limited frequency bandwidth. Complex frequency modulation and data acquisition equipment is required, making the system costly.

Based on the above description, SDM, PDM, and FDM are considered three relatively effective and practical methods for suppressing the fading noise. Although each of these methods has certain limitations, the optimal trade-off between the complexity of hardware and performance in various application scenarios can be achieved by exploring reasonable scheme designs and algorithm combinations. For different application requirements, optimal fading noise suppression can be obtained within the acceptable cost range. In this paper, a fading suppression method for Φ-OTDR systems based on multi-domain multiplexing (MDM) is proposed. We discuss the fading noise suppression effects when these methods are used individually, as well as how to select the number of polarization states in PDM, the number of frequencies in FDM, and the spatial weighting intervals in SDM in multi-domain multiplexing, to achieve the lowest hardware cost and the best fading suppression effect that meet the application requirements.

## 2. Multi-Domain Multiplexing Principle

In a heterodyne Φ-OTDR system, fluctuations of the RBS are randomly variable, and the electric field of the RBS is as follows:

(1) $$E_{RBS}(t,z)=\sqrt{2a_{s}(t,z)SWe^{-2az}}E_{s}\exp\left[\omega_{s}\cdot \frac{2z}{v_{g}}+\varphi_{s}(t,z)\right]$$

where *W* is the fiber length corresponding to the pulse width τ of the probe pulse light, a is the fiber attenuation coefficient, $a_{s}\left(t,z\right)$ is the RBS scattering coefficient whose value varies with position, S is the capture factor of the RBS, and $\omega_{s}=\omega_{0}+∆\omega$, where $\omega_{0}$ is the frequency of the laser, ∆ω is the frequency shift, $\varphi_{s}(t,z)$ is the phase, and $v_{g}$ is the speed of light in the optical fiber.

The above model is based on the complete matching of the polarization state of the RBS signal and that of the local light. In fact, due to the fiber birefringence effect, the polarization state of light evolves continuously along the length of the fiber, showing complex variation characteristics. To analyze the influence of the polarization state, the optical signal is decomposed into two mutually orthogonal components, the P-state and the S-state, through the Jones matrix:

(2) $$\left(\begin{array}{c} E_{s1}(t)\\ E_{p1}(t) \end{array}\right)=E_{s}\exp\left[j(\omega_{s}t+\varphi_{s})\right]\left(\begin{array}{c} \cos\theta_{s}\cos\varepsilon_{s}-j\sin\theta_{s}\sin\varepsilon_{s}\\ \sin\theta_{s}\cos\varepsilon_{s}+j\cos\theta_{s}\sin\varepsilon_{s} \end{array}\right)$$

(3) $$\left(\begin{array}{c} E_{s2}(t)\\ E_{p2}(t) \end{array}\right)=E_{l}\exp\left[j(\omega_{l}t+\varphi_{l})\right]\left(\begin{array}{c} \cos\theta_{l}\cos\varepsilon_{l}-j\sin\theta_{l}\sin\varepsilon_{l}\\ \sin\theta_{l}\cos\varepsilon_{l}+j\cos\theta_{l}\sin\varepsilon_{l} \end{array}\right)$$

where $\omega_{l}=∆\omega$, $\varphi_{l}$ is the phase of local light, $\theta_{s}$ and $\theta_{l}$ are the azimuth angles of the RBS signal and the local light, respectively, and $\varepsilon_{s}$ and $\varepsilon_{l}$ are the ellipticity angles of the RBS signal and the local light, respectively. When the probe light and the local light pass through the analyzer with an angle of θ to the *x*-axis, the output light intensity is as follows:

(4) $$I(t)=I_{0}(t)+E_{s}(t)E_{l}(t)\sqrt{a_{\theta }^{2}+b_{\theta }^{2}}\cos\left[\varphi (t)+\arctan\left(\frac{b_{\theta }}{a_{\theta }}\right)\right]$$

where φ(t) is $\omega_{s}-\omega_{l}$, $a_{\theta }$ and $b_{\theta }$ can be written as follows:

(5) $$a_{\theta }=\cos(\theta_{s}-\theta_{l})\cos(\varepsilon_{s}-\varepsilon_{l})+\cos(\theta_{s}+\theta_{l}-2\theta )\cos(\varepsilon_{s}+\varepsilon_{l})$$

(6) $$b_{\theta }=\sin(\theta_{s}-\theta_{l})\sin(\varepsilon_{s}-\varepsilon_{l})+\sin(\theta_{s}+\theta_{l}-2\theta )\sin(\varepsilon_{s}+\varepsilon_{l})$$

The visibility of the detection signal is as follows:

(7) $$V=\frac{\sqrt{a_{\theta }^{2}+b_{\theta }^{2}}}{2}$$

When the probe pulse light propagates in the optical fiber, it is affected by the random changes in fiber birefringence. As a result, the polarization state of the RBS signal cannot maintain an optimal match with the local light. That is, *θ* is different along the fiber link, which leads to different visibilities of the signals detected by the detector. In the worst scenario, when the polarization state of the RBS signal is completely orthogonal to that of the local light, the detector cannot output an effective signal.

The fading noise can cause the IF signal to enter a low amplitude range, which causes fading and then results in low accuracy in the phase demodulation result. After IQ demodulation of the amplitude and phase demodulation of the IF signal, the phase of the IF signal with noise can be obtained as follows [14]:

(8) $$\varphi (t,z)=\tan^{-1}\frac{A_{SNR}\sin(\varphi_{s}(t,z))+\sin(\varphi_{n}(t,z))}{A_{SNR}\cos(\varphi_{s}(t,z))+\cos(\varphi_{n}(t,z))}$$

where $A_{SNR}$ is the SNR of the IF signal, $A_{SNR}=A_{IF}/A_{n}$, where $A_{n}$ is the amplitude noise, and $\varphi_{n}\left(t,z\right)$ is the phase noise. $A_{SNR}$ can be used as a criterion for phase accuracy. When $A_{SNR}$ is too low, the demodulated phase φ(t,z) has a relatively low accuracy. The higher the $A_{SNR}$, the more accurate the phase. According to Equations (1), (4), and (7), the output light intensity is simplified as follows:

(9) $$I(t,z)\propto \sqrt{2a_{s}(t,z)SWe^{-2az}}E_{s}E_{l}V\exp\left[\Delta \omega \cdot \frac{2z}{v_{g}}+\varphi_{s}(t,z)+\arctan\left(\frac{b_{\theta }}{a_{\theta }}\right)\right]$$

The factors affecting the $I\left(t,z\right)$ signal in Equation (9) are not only limited to the scattering coefficient and position, but also include *W* corresponding to the width of the probe pulse, the optical frequency Δω, and the visibility of the detection signal *V*. Firstly, at different positions *z*, $I\left(t,z\right)$ has different distributions, which implies the feasibility of spatial multiplexing in fading suppression. However, it is not the case that the larger the spatial intervals for multiplexing, the better. When *W* is relatively small, the overall amplitude of the signal is relatively small, and fading is more likely to occur, which requires multiplexing more spatial intervals to make up for it. As *W* increases, the overall value of $I\left(t,z\right)$ increases, and the probability of fading occurrence decreases, so the demand for the number of multiplexed spatial intervals also decreases. The amount of multiplexed spatial intervals and the spatial resolution should find a balance between fading suppression and other performances. Secondly, for different Δω and V, $I\left(t,z\right)$ has different distributions as well. To obtain polarization state-independent measurement channels, we can use PDM through receiving orthogonal polarization states in the polarization state domain. To obtain frequency-independent measurement channels, FDM can be employed by transmitting multifrequency probe light.

Through $A_{SNR}$ in Equation (8), the phase accuracy can be evaluated. Setting the magnitude of $A_{SNR}$ as the criterion based on previous research [14], weights are assigned to multi-domain independent measurement channels. According to the assigned weights, the final phase is synthesized through weighting. Then, fading noise is eliminated.

## 3. Schematic of the Proposed Multi-Domain Multiplexing System

**Figure 1:**
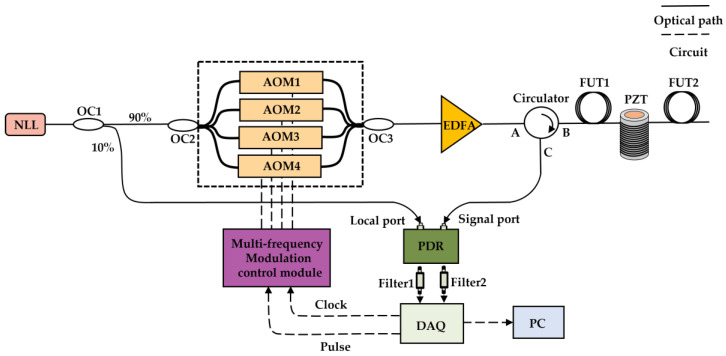
Schematic of the proposed MDM system.

In the previous section, the MDM principle was introduced. In this section, we describe a test experiment using an MDM Φ-OTDR system, and the schematic diagram of it is shown in Figure 1.

The principle of this system shown in Figure 1 is as follows. A narrow linewidth laser (NLL) emits polarization-maintaining continuous-wave (CW) laser light with a wavelength of 1550.12 nm, a linewidth of 3 kHz, and a power of 13.1 dBm. After passing through a 1 × 2 polarization-maintaining optical coupler (OC1) with a splitting ratio of 90:10, 90% of the optical power enters a 1 × 4 polarization-maintaining fiber coupler with the same splitting ratio and is injected into acousto-optic modulator (AOM) crystals with frequency shifts of 80 MHz, 100 MHz, 120 MHz, and 150 MHz as the probe light, respectively. The remaining 10% of the optical power enters a polarization diversity receiver (PDR) as the local light. The four AOM crystals are then combined using a 4 × 1 polarization-maintaining fiber coupler with the same splitting ratio to form multifrequency probe pulse lights, which is manufactured by Nanjing Fiber Technology Co., Ltd. in Nanjing, China. These pulse lights are amplified by an erbium-doped fiber amplifier (EDFA) to compensate for insertion losses and enhance pulse energy. These amplified probe pulse lights enter the circulator (CIR) through port A, and then enter the fiber under test (FUT) through port B. The RBS signal from the FUT enters the polarization diversity receiver (PDR) with a bandwidth of 300 MHz through port C of the CIR. The PDR acts as an optical receiver, receiving the RBS and local light and outputting dual IF electrical signals with polarization states P (P state) and S (S state). The IF signals of the P state and the S state are filtered through Filter 1 and Filter 2. Filter 1 and Filter 2 are exactly the same. The center frequency of the filter is 115 MHz. The in-band flatness within the passband ranging from 67 MHz to 163 MHz is less than 0.8 dB, and the out-of-band rejection ratio at the frequencies of 50 MHz and 184 MHz exceeds 50 dB. Hence, the signals with frequencies of 80 MHz, 100 MHz, 120 MHz, and 150 MHz are within the passband, and the wide-spectrum out-of-band noise is suppressed to improve the signal-to-noise ratio of the intermediate-frequency signal, which is conducive to improving the accuracy of the demodulated phase.

**Figure 2:**
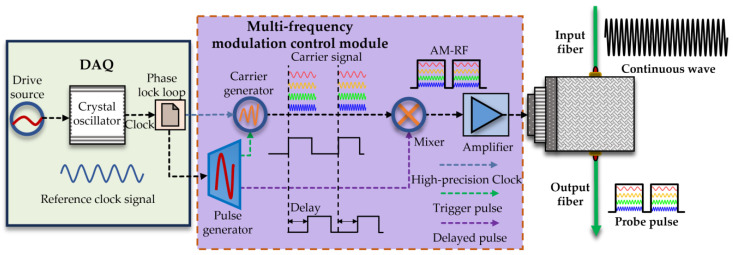
The generation process of the multifrequency probe pulse light.

In Figure 2, the generation process of the multifrequency probe pulse light is illustrated further. A 10 MHz clock signal and pulse are generated by the DAQ and synchronized with the multifrequency modulation control module. As shown in Figure 2, a highly stable high-precision 10 MHz reference clock is obtained after the crystal oscillator clock passes through the phase-locked loop (PLL), and a high-precision timing pulse is obtained through frequency division. The high-precision clock signal output by the PLL is input into the carrier generation circuit, and the pulse signal is input into the pulse generation circuit. The pulse generation circuit counts according to the output clock of the phase-locked loop to generate a trigger signal and a delayed pulse. The carrier generation circuit generates continuous sinusoidal carrier signals with frequencies of 80 MHz, 100 MHz, 120 MHz, and 150 MHz according to this trigger signal. The carrier signal and the delayed pulse are mixed using a mixer to generate an electrical pulse signal in the radio frequency band, which is then amplified by a power amplifier to finally obtain an amplitude-modulated radio frequency (AM-RF) signal. The AM-RF signal is used to drive the acousto-optic modulators (AOMs) to modulate the continuous light wave into a probe pulse light with multiple frequency shifts. Through this modulation method, the data sampling clock of the DAQ is highly synchronized with the generation of the multifrequency modulation probe pulse light. It can effectively eliminate the noise interference caused by clock asynchrony, reduce the phase noise of the system, and improve the signal-to-noise ratio of the intermediate-frequency signal.

For the data capture by the DAQ, the maximum frequency of the intermediate-frequency signal output at the detector end is 150 MHz. According to the Nyquist sampling theorem, the sampling rate of the data acquisition card should reach at least twice the maximum frequency, that is, $f_{s}$ = 300 MSa/s, to reconstruct this intermediate-frequency signal. In addition to meeting the Nyquist sampling theorem, to ensure a good reconstruction of the intermediate-frequency signal, the sampling rate should be an integral multiple of the carrier frequency of the intermediate-frequency signal. For the intermediate-frequency signals with frequencies of 80 MHz, 100 MHz, 120 MHz, and 150 MHz, the sampling rate of the data acquisition card should preferably be selected as the common multiple of these intermediate-frequency signal frequencies. Therefore, the sampling rate can be selected as 600 MSa/s, which can meet the requirement of integral multiple sampling for the intermediate-frequency signals of 100 MHz, 120 MHz, and 150 MHz. For the intermediate-frequency signal of 80 MHz, although the sampling rate does not satisfy the integral multiple relationship, since the sampling rate is several times the signal frequency, it can still ensure that sufficient sampling points are obtained for each signal period, thus ensuring the integrity of the signal.

**Figure 3:**
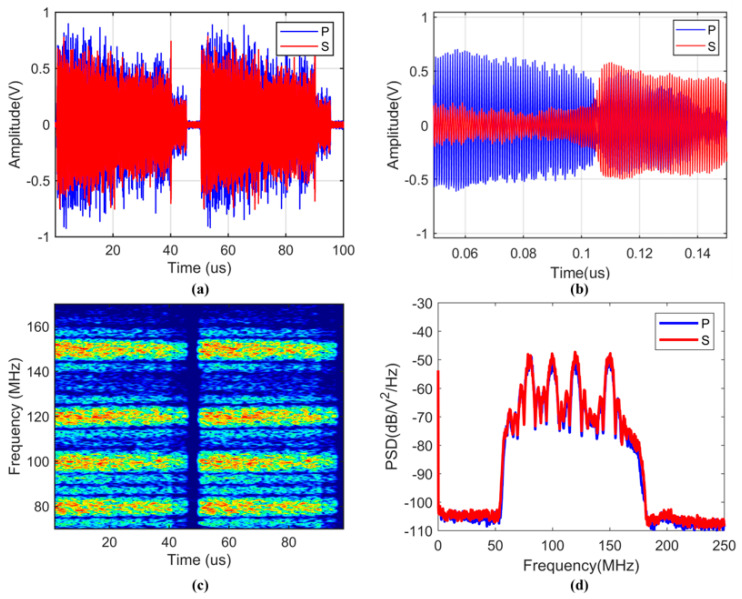
(**a**) Waveform diagram of the IF signal; (**b**) local waveform diagram of the IF signal; (**c**) STFT spectrum diagram of the IF signal; (**d**) power spectrum diagram of the IF signal.

Figure 3a shows the IF signal waveforms of the P and S states, and the signal energy gradually decays as the fiber distance increases. Figure 3b shows the local waveform of the IF signal, and the P state and the S state are roughly complementary. Figure 3c shows the short-time Fourier transform (STFT) spectrum of the IF signal. The yellow area indicates that the IF signal contains energy peaks at four frequencies of 150 MHz, 120 MHz, 100 MHz, and 80 MHz, and these frequencies are highly overlapped in time. Figure 3d shows the power spectrum of the IF signal. The four frequency points of 150 MHz, 120 MHz, 100 MHz, and 80 MHz have the same peak intensity. The main peaks all have a high SNR and a narrow spectral width, and the side lobes are suppressed well. Therefore, the IF signal shows good spectral characteristics.

## 4. Experimental Results and Analysis

In this section, the effects of the spatial weighting, PDM, and FDM optimal methods on fading noise suppression are discussed.

### 4.1. Spatial Weighting

Based on the previous weighting method, with spatial weighting intervals being 0.25–2 of the gauge length (GL), the improvement of the SNR and the deterioration of the spatial resolution caused by the amount of weighting intervals were discussed. The GL and the SNR are defined as Equations (10) and (11), respectively:

(10) $$SNR=10log_{10}(\frac{P_{s}}{P_{n}})$$

where $P_{s}$ is the power of the signal and $P_{n}$ is the power of the noise.

(11) $$GL=\frac{v_{g}\cdot \tau }{2}$$

**Figure 4:**
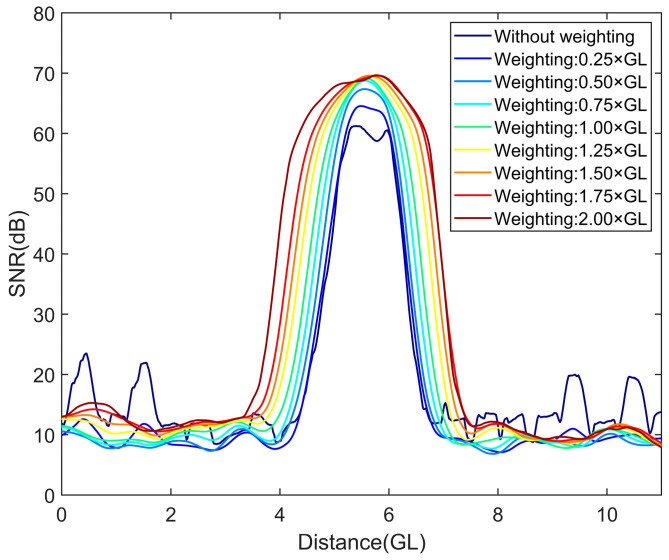
SNR and spatial distribution under different weighting intervals.

**Figure 5:**
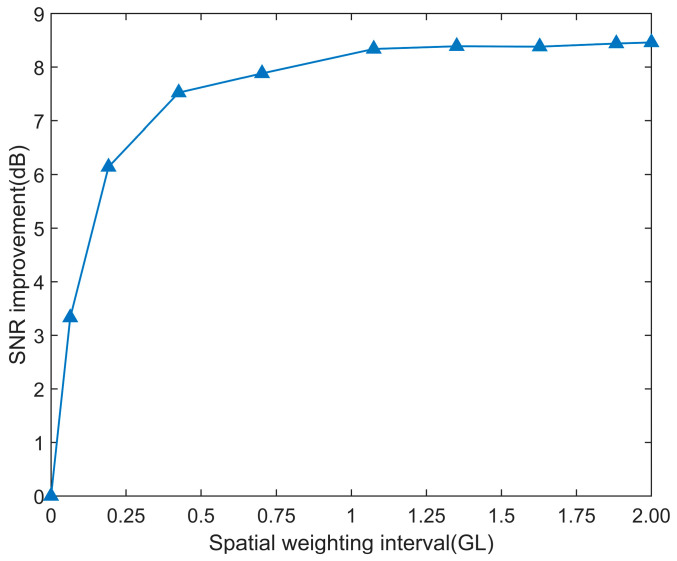
SNR improvement and RMS under different weighting intervals.

In Figure 4, as the number of spatial weighting intervals increases, the SNR of the disturbance signal is enhanced, the spatial distribution of the disturbance is obviously broadened, and the spatial resolution decreases. The increase in the weighting length has an impact on the improvement of the SNR, as shown in Figure 5.

In Figure 5, when the number of spatial weighting intervals increases from 0 to GL, the SNR is improved by approximately 8 dB. However, when the spatial weighting intervals exceed GL, even if the amount of spatial weighting intervals is further increased, the improvement in the SNR is negligible. Therefore, for the selection of the number of spatial weighting intervals, it is better to select the corresponding spatial weighting length no larger than the GL.

### 4.2. PDM, FDM, and Spatial Weighting

In this section, the contributions of PDM and FDM to the suppression of fading noise are compared, and we show this effect of fading suppression through waterfall charts and the root mean square of the demodulated phase of optical fiber. The length of the optical fiber connected is 4 km for FUT1 and 200 m for FUT2, the pulse repetition frequency is set to 20 kHz with a pulse width of 200 ns, and a sinusoidal signal of 30 Hz and 0.2 V is applied using PZT.

**Figure 6:**
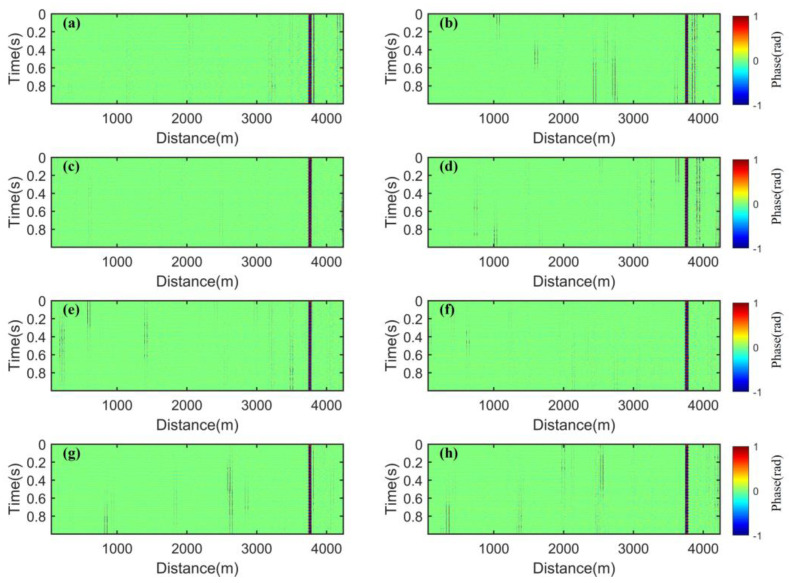
Waterfall charts of the optical fiber without disturbance: (**a**) 80 MHz, P state; (**b**) 80 MHz, S state; (**c**) 100 MHz, P state; (**d**) 100 MHz, S state; (**e**) 120 MHz, P state; (**f**) 120 MHz, S state; (**g**) 150 MHz, P state; and (**h**) 150 MHz, S state.

**Figure 7:**
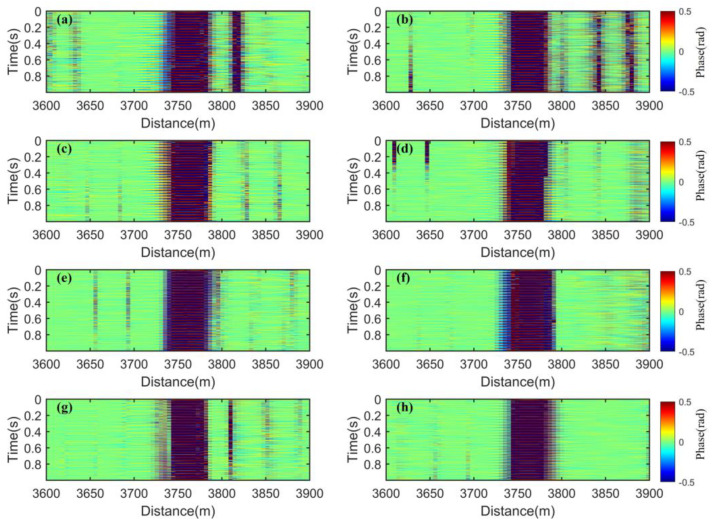
Waterfall charts of the optical fiber with a disturbance event: (**a**) 80 MHz, P state; (**b**) 80 MHz, S state; (**c**) 100 MHz, P state; (**d**) 100 MHz, S state; (**e**) 120 MHz, P state; (**f**) 120 MHz, S state; (**g**) 150 MHz, P state; (**h**) 150 MHz, S state.

As shown in Figure 6 and Figure 7, the waterfall charts display the phase demodulation waterfall diagrams of the P state and S state at frequencies of 80 MHz, 100 MHz, 120 MHz, and 150 MHz. As shown in Figure 6, fading events of varying degrees are shown at the positions of the optical fiber where no disturbance is applied. As shown in Figure 7, the applied disturbance events are distorted due to the influence of fading, and the spatiotemporal continuity is disrupted. This means there may be false alarms and a decrease in the accuracy of disturbance event reconstruction, which is not conducive to the engineering application of the Φ-OTDR system. Especially in Figure 6b, the influence of fading is particularly evident.

**Figure 8:**
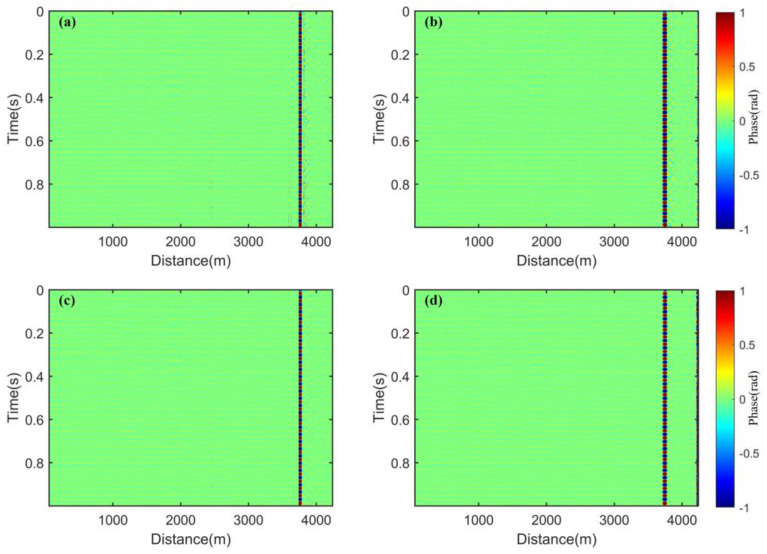
Waterfall charts of the optical fiber: (**a**) PDM with the P state and the S state, (**b**) spatial weighting with a weighting interval of GL, (**c**) FDM with 4 frequencies, (**d**) a combination of the first three methods.

We separately optimize the demodulated phases in Figure 7b using PDM with the P state and the S state, spatial weighting with a weighting interval of GL, FDM with 4 frequencies, and a combination of the first three methods. The results are shown in Figure 8.

**Figure 9:**
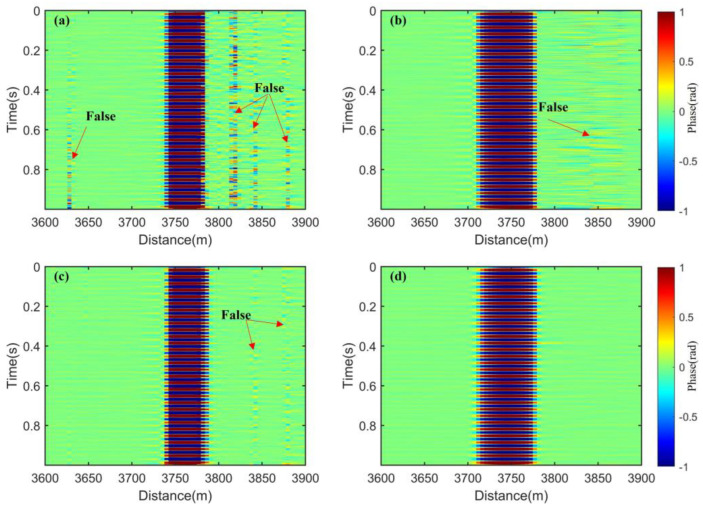
Waterfall charts of the optical fiber with a disturbance event: (**a**) PDM with the P state and the S state, (**b**) spatial weighting with a weighting interval of GL, (**c**) FDM with 4 frequencies, (**d**) a combination of the first three methods.

As shown in Figure 8, when PDM is used alone, the fading noise is greatly suppressed, but obvious residues can still be observed near the disturbance event. To clearly demonstrate the effectiveness of these four methods in suppressing the fading noise, we focused on observing the area ca. 3600–3900 m around the disturbance event, which is shown in Figure 9.

**Figure 10:**
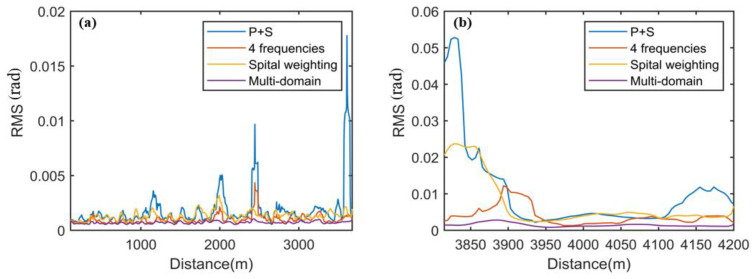
Distance distribution of the RMS: (**a**) the RMS distance distribution of the quiet area before the disturbance event; (**b**) the RMS distance distribution of the quiet area after the disturbance event.

In Figure 9, near the disturbance event in Figure 9a–c, it is clearly observable that there are still a few residual noises, which lead to false events. However, in Figure 9d, this phenomenon is well-eliminated. This indicates that the fading noise can be more comprehensively suppressed by combining PDM, FDM, and spatial weighting. We use the RMS to quantitatively evaluate the fading suppression effects of these four methods on the spatial regions where no active disturbances have been applied. That is shown in Figure 10. When the four methods are used separately, the RMS value of PDM is the largest, indicating the worst suppression effect on fading noise. The RMS value of MDM is the smallest, dropping below 0.005, showing the best fading suppression effect.

The above results and analyses indicate that it is difficult to comprehensively combat fading noise by using a single method. Therefore, MDM is essential for combating fading noise. However, the abovementioned MDM method is based on the performance limits of these methods. In practice, it is not always necessary to use exactly four frequencies, nor is it always necessary to set the weighting intervals to GL. We are eager to find a combination that minimizes costs and achieves the best fading suppression. To this end, we try our best to exhaust all combinations of the parameters of these methods in multi-domain multiplexing, including the number of polarization states in PDM, the number of frequencies in FDM, and the intervals of spatial weighting.

**Figure 11:**
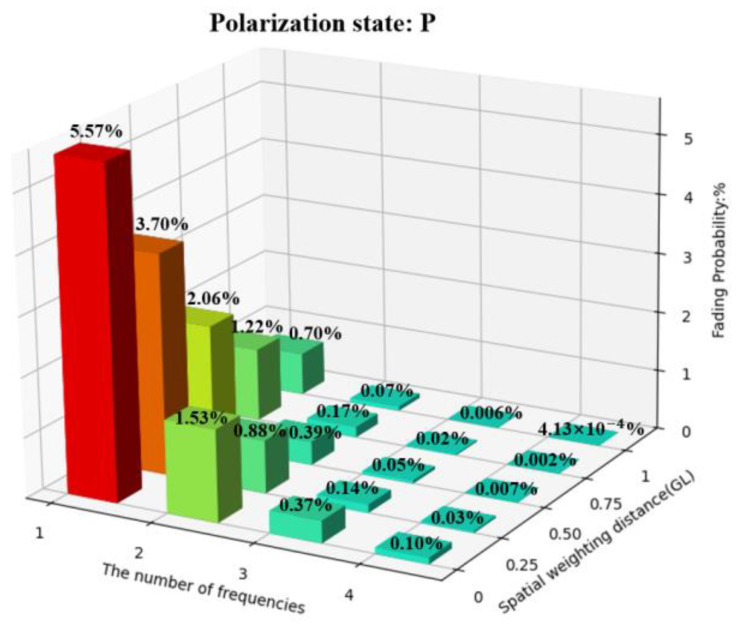
Fading probabilities of all combinations in the P state.

**Figure 12:**
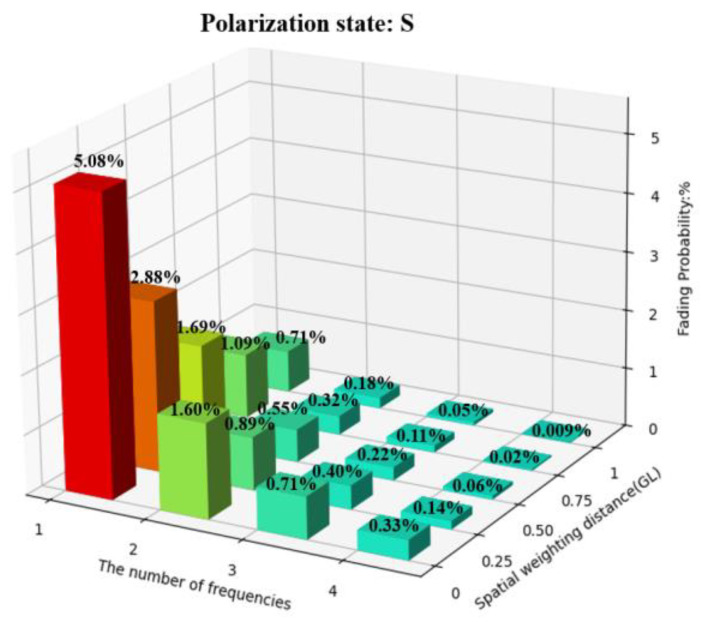
Fading probabilities of all combinations in the S state.

**Figure 13:**
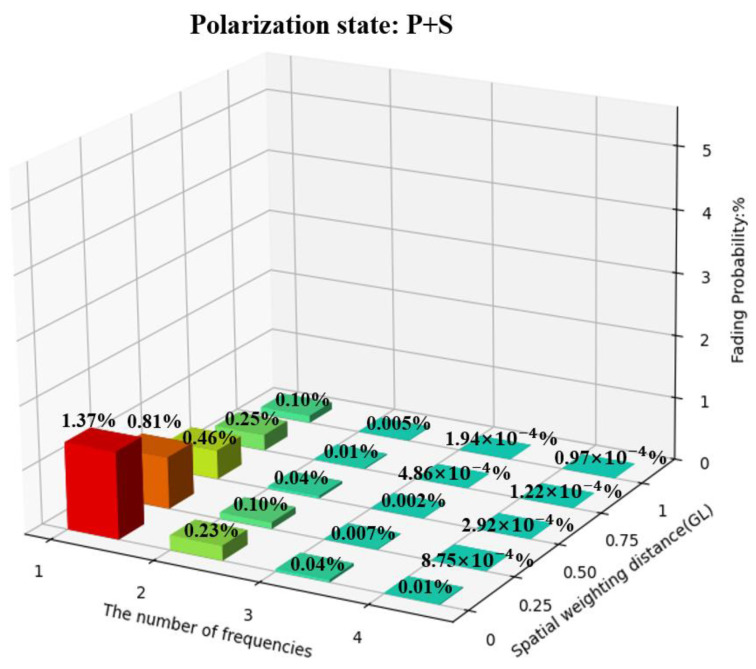
The fading probabilities of all combinations in the P and S states.

To quantitatively evaluate fading resistance capabilities of these combinations, we present the fading resistance performance of these combinations in the form of three-dimensional bar charts. The specific situations are divided into a single P state, a single S state, and the combination of both P and S. The coordinate axes on the bottom surface of the figure represent the number of frequencies and the amount of spatial weighting intervals, respectively, and the *z*-axis represents the probability of fading occurrence. The number of frequencies is 1, 2, 3, and 4, respectively, and the spatial weighting intervals are 0, 0.25 GL, 0.5 GL, 0.75 GL, and GL, respectively. The RMS value for fading is defined as half of the value in previous studies [14]. The probabilities of fading occurrence for all combinations are shown in Figure 11, Figure 12 and Figure 13.

In Figure 11 and Figure 12, to achieve a 1% fading probability, when the number of frequencies is 1, the intervals of spatial weighting should be at least GL, which means sacrificing a relatively large spatial resolution. When the number of frequencies is 2, the intervals of spatial weighting should be at least 0.25 GL. When the number of frequencies is greater than or equal to 3, a less than 1% fading probability can be achieved without spatial weighting.

In Figure 13, after adding PDM in the P and S states, a 1% fading probability can be achieved using 2-frequency FDM without a decline in spatial resolution. Using FDM with more than 3 frequencies can achieve a 0.01% fading probability without a decline in spatial resolution. If the decline of spatial resolution can be accepted, a 0.01% fading probability can be achieved with only 2-frequency FDM and a spatial weighting interval of 0.25 GL.

The above analysis results show that it is feasible to achieve a balance between the performance and hardware complexity of different application conditions through MDM. This provides a guiding solution for the anti-fading of Φ-OTDR. In engineering applications, the optimized design for fading suppression can be flexibly carried out, which broadens the scope of engineering applications of Φ-OTDR.

In the future, further research can focus on the design strategies of Φ-OTDR systems. It is necessary to reduce the system cost and complexity while ensuring the fading suppression effect to improve the overall performance of the system. There is no need to use a 4-frequency FDM as in this paper. Instead, according to the actual application requirements, the optimal combination of hardware and algorithms in terms of cost and performance should be selected.

## 5. Conclusions

In this paper, we propose a fading suppression method for Φ-OTDR systems based on MDM, which fuses the advantages of spatial weighting, PDM, and FDM, obtaining the fading-free demodulated phase. By means of parameter selection and optimized combination of the domain in MDM, an optimal solution for anti-fading can be achieved between performance and hardware complexity, which promotes the development of the fading suppression technology of Φ-OTDR. This provides a guide for the high-fidelity sound field sensing of Φ-OTDR.

## Data Availability

All original data, vibration data, and code will be made available upon request to the corresponding author’s email with appropriate justification.

## Abbreviations

The following abbreviations are used in this manuscript:

Φ-OTDR	Phase-sensitive optical time domain reflectometry
RBS	Rayleigh backscattered signal
IF	Intermediate frequency
SNR	Signal-to-noise ratio
SDM	Spatial domain multiplexing
PDM	Polarization domain multiplexing
FDM	Frequency domain multiplexing
CNR	Carrier-to-noise ratio
OSPP	Orthogonal SOP pulse pair
MDM	Multi-domain multiplexing
NLL	Narrow linewidth laser
CW	Continuous wave
OC	Optical coupler
AOM	Acousto-optic modulator
PDR	Polarization diversity receiver
DAQ	Data acquisition card
EDFA	Erbium-doped fiber amplifier
CIR	Circulator
FUT	Fiber under test
PZT	Piezoelectric ceramic transducer
AWG	Arbitrary waveform generator
STFT	Short-time Fourier transform
GL	Gauge length
RMS	Root mean square
